# The effect of a postoperative quality improvement program on outcomes in colorectal surgery in a community hospital

**DOI:** 10.1007/s00384-016-2619-1

**Published:** 2016-07-06

**Authors:** C. C. M. Marres, A. W. H. van de Ven, P. C. M. Verbeek, S. van Dieren, W. A. Bemelman, C. J. Buskens

**Affiliations:** 1 Department of Surgery, Flevoziekenhuis, Hospitaalweg 1, 1315 RA Almere, the Netherlands; 2 Department of Surgery, Academisch Medisch Centrum, Amsterdam, the Netherlands

**Keywords:** Colorectal surgery, Colorectal resection, Oncology, Quality improvement, Complications

## Abstract

**Purpose:**

The aim of this study was to evaluate whether implementation of a comprehensive quality improvement program was associated with improved outcomes in patients undergoing oncological colorectal surgery in a non-academic, non-referral community hospital.

**Methods:**

The quality improvement program (QIP) was introduced in January 2011 and consisted of the following interventions: (1) avoidance of postoperative nonsteriodal anti-inflammatory drugs; (2) normovolemia was pursued pre- and postoperatively; (3) non-resectional surgery if possible, in patients over 80 with ASA 3 or 4 classification; and (4) a standardized, postoperative surveillance protocol was introduced, with CRP determination day 2 and 4, and if necessary subsequent abdominal CT with rectal contrast to reduce delay in diagnosis of complications. From a prospectively maintained database of 488 patients undergoing colorectal surgery between 2009 and 2014, postoperative outcomes of patients operated before and after implementation of the program were compared.

**Results:**

The severe complication rate (Clavien-Dindo >3b) decreased significantly (25.0 vs. 13.7 %; *p* < .001) after implementation of the QIP program. The mortality rate dropped from 8.7 to 2.6 % (*p* = .003). The percentage of anastomotic leakage was 9.6% before QIP implementation and 4.2% after (*p* = .013). Median length of hospital stay decreased from 9 (IQR 5–19) to 7 days (IQR 4–12) (*p* < .001). Multivariate analyses showed that surgery after implementation of the program was a strong independent predictor for less major complications (OR 0.54, 95 % CI 0.32–0.88).

**Conclusions:**

A significant decrease in major complications and mortality was observed after introduction of a relative simple quality improvement program.

## Introduction

Oncological colorectal surgery carries a moderately high risk for morbidity and mortality. In the literature, the incidence of major complications range from 20 to 35 % and the 30-day mortality rate from 2 to 9 % [1–3].

During a national clinical audit on oncological colorectal surgery in 2010 in The Netherlands, a relatively high complication and mortality rate was seen in our hospital compared to overall national outcomes [4]. Therefore, after careful re-evaluation of the auditing results and an extensive literature analyses for factors influencing colorectal complications, a comprehensive quality improvement program was composed and introduced in January 2011 to decrease complications and mortality. The aim of this study was to evaluate whether implementation of this program was associated with improved outcomes in patients undergoing oncological colorectal surgery in a non-academic, non-referral community hospital.

## Methods

### Study design

A cohort study was designed from a prospectively maintained database of patients undergoing an oncological colorectal resection between 2009 and 2014. Patients operated before and after implementation of the QIP program were compared. The institutional review board approved this study.

### Establishment of the quality improvement program (QIP)

A systematic literature search was performed at the end of 2010 using PubMed and Embase. Factors possibly influencing colorectal surgery outcomes were identified and were summarized in Table 1. The literature for all these different factors was compared to our treatment protocol at the time. Each factor was analyzed to see if there was room for improvement in our protocol. Some factors were simply not feasible or applicable in our hospital, such as routinely intra-operative leak testing. In other factors, our treatment protocol was in concordance with the optimal treatment according to the literature. Eventually, four factors remained, which seemed suitable for improvement and the following interventions were implemented in our postoperative protocol: Firstly, the use of postoperative nonsteriodal anti-inflammatory drugs was stopped [5–7]. Secondly, normovolemia was pursued pre- and post-operatively [8–10]. Furthermore, acute resections (with immediate anastomosis) in patients over 80 years with ASA 3 or 4 classification were avoided [11]. In case a frail, high risk patient presented with acute, colonic obstruction, the aim was to manage the obstruction with a colostomy in the acute phase and only perform resection of the tumor in elective setting after optimization of the patient [12–14]. Finally, a standardized postoperative surveillance protocol to reduce delay in diagnosis of complications was introduced; standardized serum C-reactive protein (CRP) level determination on day 2 and 4, if day 4 CRP showed an increase of 50 or an elevation above 200 mg/L, an abdominal CT with rectal contrast was performed [15, 16].

**Table 1:** Factors that might have impact on postoperative complications

Factors that cannot be changed	Possible association with increased risk postoperative complications
Age	>70 years [1–3]
Gender	Male [1–3]
ASA classification	Grade 3 or 4 [1–3]
Urgency operation	Emergency surgery [1–3]
Type of resection	Rectal excision [1–3]
Stage of cancer	Stage IV [1–3]
Obesity	BMI >25 [1–3]
Changeable factors	
Preoperative mechanical bowel preparation	
Experience of surgeon/specialized	
Laparoscopic or open procedure	
Technique anastomose	
Intra-operative leak testing	
Protective stoma	
Pelvic drain	
Operating time, operative strategy	
Prophylactic antibiotics	
Thrombose prophylactic	
Postoperative analgesia (NSAID, opioids)	
Fast track recovery protocols	
Biomarkers for early diagnosis of complication	

With respect to other factors possibly influencing outcomes, the perioperative approach remained unchanged during the timeframe of the study. Postoperatively, patients were managed according to ERAS fast track protocol [17]. Preparation for surgery was the same for all patients. No bowel preparation was given, except for patients undergoing left colonic and rectal resections [18, 19]. All colorectal resections were performed by or under supervision of specialized colorectal surgeons [20]. Anastomoses were made with a stapler device. In patients who underwent a hemicolectomy, a side-to-side anastomose was established and a side-to-end in patients who underwent sigmoid and rectal resections, through the low tie technique (ligation at the level of the superior rectal artery, just caudally to the origin of the left colic artery) [21]. Intra-operative leak testing was only done in very high suspicious situations in low rectal anastomoses through an air leak test. Defunctioning stomas and drain tubes were provided subjective to the surgeon’s opinion of the quality of the anastomoses during surgery. Antibiotic prophylaxis was given trough cefazolin 1000 mg plus metronidazole 500 mg intravenously 30 min prior to surgery. If the operation took longer than 3 h, this was repeated. All patients received low-molecular-weight heparin daily during admission to prevent venous thromboembolism [22].

### Study population and data collection

A cohort study was designed from a prospectively maintained database of patients undergoing an oncological colorectal resection in our institution between 2009 and 2014. Patients who underwent transanal endoscopic microsurgery were excluded. The medical records of 484 patients were reviewed. The prospectively maintained database with patient characteristics was supplemented with information on CRP levels, timing, and outcome for postoperative CT scans of the abdomen and following interventions. Patients were divided into two cohorts for comparison: those operated before and after implementation of the QIP program in January 2011.

### Outcome measures

The primary outcome measure of interest was major complication. Complications were graded according to the Clavien-Dindo classification [23], and severe complications being defined as grade 3b or higher. Secondary outcome parameters were all postoperative complications, 30-day mortality, failure-to-rescue [24], length of hospital stay, and timing of CT scans and of re-interventions. Failure-to-rescue rates were defined as mortality among patients with severe complications.

### Statistical analysis

Statistical analysis was performed using IBM SPSS Statisitics for Windows, version 22.0 (IBM corp., Chicago, IL). Continuous variables were presented as mean values with a standard deviation (SD) or as median values with an interquartile range (IQR) according to the distribution (Kolmogorov-Smirnov and Sapiro-Wilk test). Discrete variables were presented as counts and percentages. Categorical data were compared between groups using the *χ*^2^ test, and continuous data were compared using the independent samples *t* test or Mann-Whitney *U* test. Univariate and stepwise multivariate logistic regression models were used to examine the association between the QIP program implementation and severe postoperative complications while adjusting for important potentially confounding variables. The limited number of events (from a statistical point of view) meant that only a restricted number of possible confounders could be examined. Therefore, variables with multiple categories were recorded into dichotomous variables by combining categories with a comparable prognosis (ASA I/II vs. III/IV, radicality R0 vs. R1 and R2, and Dukes A-B vs. C-D).

Clinical important variables based on literature were included in the univariate and multivariate models, as well as all variables with a univariate *p* value<.20. A two-tailed *p* value of <.05 was considered statistically significant.

## Results

### Patient demographics

There were 265 (54.8%) men and 219 (42.2%) women analyzed in this study with a mean age of 66 years (range 28–89). Relevant patient demographics were summarized in Table 2. Univariate analyses demonstrated no significant differences between the two cohorts concerning age, BMI, sex, ASA classification, tumor stage, stoma or no stoma, and urgency of the operation. There were significantly more minimally invasive procedures in the cohort operated after the QIP program implementation, as expected due to the longitudinal setting of this study. Also, a significant difference in type of resection between the two cohorts was found, with more radical resections in the later patient group. These differences were taken into account in the multivariate analyses. Missing data were for every variable less than 5 %, and therefore, there was no imputation of missing data.

**Table 2:** Patient demographics

Variables	Pre-implementation *N* (%)^a^	Post-implementation *N* (%)^a^	Total *N* (%)^a^	*p* value
Age				.283^b^
Average (SD)	67 (13)	66 (11)	66 (12)	
Range	30–96	30–90	28–89	
Sex				.545^c^
Female	81 (47.1)	138 (44.2)	219 (45.2)	
Male	91 (25.9)	174 (55.8)	265 (54.8)	
BMI				.989^b^
Average (SD)	26.3 (4.2)	26.3 (4.5)	26.3 (4.4)	
Range	16.5–45.3	16.2–50.2	16.2–50.2	
ASA				.246^c^
ASA 1 or 2	130 (75.6)	251 (80.4)	381 (78.7)	
ASA 3 or 4	42 (24.4)	61 (19.6)	103 (21.3)	
Type of operation				.006^c^
Right hemicolectomy	49 (28.5)	109 (34.9)	158 (32.6)	
Left hemicolectomy	12 (7.0)	24 (7.7)	36 (7.4)	
Sigmoidectomy/LAR	87 (50.6)	139 (44.6)	226 (46.7)	
Colectomy	2 (1.2)	18 (5.8)	20 (4.1)	
APR	12 (7.0)	19 (6.1)	31 (6.4)	
Other	10 (5.8)	3 (1.0)	13 (2.7)	
Approach				<.001^c^
Open	41 (23.8)	27 (8.7)	68 (14.0)	
Scopic	131 (76.2)	285 (91.3)	416 (86.0)	
Stoma				.370^c^
No stoma	114 (66.3)	230 (73.7)	344 (71.1)	
Loop ileostomy	31 (18.1)	51 (16.4)	82 (16.9)	
End colostomy	27 (16.7)	31 (10.0)	58 (12.0)	
Urgency				.231^c^
Elective	150 (87.2)	283 (90.7)	433 (89.5)	
Emergency	22 (14.7)	29 (9.3)	51 (10.5)	
Dukes classification				.735^c^
Dukes’ A	0 (0)	3 (1.0)	127 (26.2)	
Dukes’ B	42 (24.4)	82 (26.3)	166 (34.3)	
Dukes’ C	54 (31.4)	102 (32.7)	142 (29.3)	
Dukes’ D	3 (1.7)	7 (2.2)	45 (9.3)	
Resection status				.002^c^
R0	155 (90.1)	308 (98.7)	463 (96.9)	
R1	10 (5.8)	2 (0.6)	12 (2.5)	
R2	1 (0.6)	2 (0.6)	3 (0.6)	
Total	172 (100)	312 (100)	484 (100)	

^a^Unless otherwise stated in the first column

^b^Mann-Whitney *U* test

^c^Chi-square test

### Outcome

The severe complication rate (Clavien-Dindo >3b) in the first cohort was 25.0 %, which significantly decreased to 13.7 % (*p* = .001) after implementation of the quality program. The mortality rate decreased from 8.7 to 2.6 % (*p* = .003). The percentage of anastomotic leakage was 9.8 % before and 4.2 % after 2011 (*p* = .013). Median length of hospital stay before implementation of the program was median 9 (IQR 5–19) and after implementation 7 (IQR 4–12) (*p* < .001). The minor complication rate (Clavien-Dindo <3b) was not significantly different in the two cohorts (respectively 24.4 and 25.3 %) (Table 3).

**Table 3:** Outcomes

	Pre-implementation *N* (%)^a^	Post-implementation *N* (%)^a^	Total *N* (%)^a^	*p* value
Mortality	15 (8.7)	8 (2.6)	23 (4.7)	.003^c^
Complication Clavien-Dindo>3b	43 (25.0)	43 (13.7)	86 (17.1)	.001^c^
Anastomotic Leak	17 (9.8)	13 (4.2)	30 (6.4)	.013^c^
Complication Clavien-Dindo <3b	42 (24.4)	79 (25.3)	117 (24.1)	.474^c^
Number of admission days (SE)	15.7 (19.7)	10.0 (9.3)	12.0 (14.1)	<.001^b^
Number days operation—CT (SE)	9.0 (6.8)	6.0 (2.2)	6.7 (5.2)	.553^b^
Number of days operation—re-intervention (SE)	9.6 (6.8)	6.8 (2.3)	7.4 (4.5)	.546^b^

^a^Unless otherwise stated in the first column

^b^Mann-Whitney *U* test

^c^Chi-square test

### Multivariate analysis

In the multivariate analysis (Table 4), all clinically and statistically relevant (*p* < .20 in univariate models for the outcome) factors were included: elective versus emergency resection, age at time of operation, ASA classification, stoma vs. no stoma, type of resection, open or minimally invasive procedures, and the date of the operation (before or after implementation of the program). Multivariate analyses showed that beside these factors, the date of the operation was a strong independent predictor for a major complication (OR 0.53, 95 % CI 0.32–0.88).

**Table 4.** Multivariate analysis; complications Clavien-Dindo >3b

	Univariate Odds ratio	Univariate 95 % CI	*p* value	Multivariate Odds ratio	Multivariate 95 % CI	*p* value
Age	1.03	1.01–1.05	.003			ns
Sex	0.86	0.53–1.39	ns			ni
ASA 1–2 vs. 3–4	2.72	1.63–4.55	.001	2.57	1.50–4.38	.001
Type of operation	1.19	1.00–1.43	.053			ns
Open vs. scopic	0.30	0.17–0.54	<.001	0.50	0.27–0.92	.027
Emergency vs. elective	2.50	1.31–4.77	.006			ns
Before vs. after 2011	0.44	0.27–0.71	.001	0.53	0.32–0.88	.014
Stoma vs. no stoma	2.56	1.58–4.17	<.001	2.14	1.27–3.61	.004
R0 vs R1-2	0.353	0.05–2.72	ns			ni
Dukes’A-B vs. C-D	1.03	0.63–1.68	ns			ni

*ns* non significant, *ni* not included

### High risk elderly patients

Before 2011, 18 patients (10.5 % of total) undergoing surgery were aged 80 years or over with an ASA 3 or 4 classification. After 2011, 20 patients (6.4 %) with these characteristics were operated upon. A primary anastomosis was made in 14 out of 18 patients (78 %), and after 2011, 11 out of 20 (55 %) received a primary anastomosis.

### CT abdomen and early intervention

The interval between operation and first CT was 9.0 days before 2011 and 6.0 days after 2011 (*p* = .553; median 7 vs. 6 days). The time until re-intervention decreased from 9.6 days before 2011 to 6.8 days after 2011 (*p* = .553; median 7 vs. 7 days) (Table 3). The number of CTs performed before and after the implementation of the QIP was comparable; before implementation of the QIP, a CT scan was performed in 29 out of 172 patients (19.8 %). Of these CTs, 11 were negative (38.0 %). After implementation of the QIP, 54 CTs were performed, which was 17.3 % of the total operated population (*n* = 312). Of these CTs, 32 were negative (59.3 %).

Failure-to-rescue rates, defined as mortality among patients with serious complications, decreased from 34.9 % before implementation of the program to 15.0 % after implementation (*p* = .039).

## Discussion

This study highlights the effect of implementing a quality improvement program in a non-academic, non-referral community hospital on surgical outcomes. A significant decrease in mortality, anastomotic leakage, and other major complications was found, with a significant decrease in length of hospital stay after implementation of the QIP program.

The interventions introduced were intended to improve our results with regard to anastomotic leakage, mortality, and other major complications. A multivariate analysis showed that patients operated before implementation of the program had a significant higher risk for a major complication with an odds ratio of 0.537. No difference was observed between the two cohorts regarding minor complication. This corroborates the conclusion that the program made a significant difference in preventing major complications.

Unfortunately, it was not possible to investigate the errors in the first two interventions after implementation of the program. Accidently, NSAID usage was not well documented, and the verifying of fluid management charts was not feasible; meaning that although it was protocolized, we could not check whether the protocol was indeed applied. Therefore, it is difficult to point out which separate intervention could be responsible for these improved results. It is unlikely that prevention of major complications in colorectal surgery is dependent on 1 or 2 factors. Rather, it is our belief that the improved results can be ascribed to a wide variety of measures [25].

The use of NSAIDS for postoperative pain management after colorectal surgery is still under debate. Currently, there is circumstantial evidence that there is a link to anastomotic leakage [26–28]. NSAIDS pre- and postoperatively as postoperative pain management did not seem an absolute necessity and instead oral opioids were used.

To this day, intra-operative fluid management remains an area of debate. The concept that fluid restriction or avoidance of fluid overload in major abdominal surgery influences perioperative outcomes, such as morbidity and length of stay, is getting more support. However, it remains unclear whether this is related to the fluid restriction, per se, or the maintenance of a state of zero fluid balance [29]. In the QIP program, a 24-h zero fluid balance was pursued versus no restriction before implementation of the program.

Another important intervention was fast re-intervention by a surveillance protocol with an abdominal CT-scan with rectal contrast was performed when infection parameters (CRP) and/or clinical symptoms where suspect of a complication. Presently, a re-intervention within 4 days after surgery is strived for in our institution. While this does not influence the major complication rate, it does decrease the subsequent sequel, resulting in shorter hospital stay and lower the failure-to-rescue rates and, by that, mortality. Strikingly, this change in protocol did not result in an increased percentage of CT’s (19.8 vs.17.3 %).

The number of elderly patients presenting with obstructive colorectal pathology is still increasing. These patients are at high risk for complications or death. A two-staged procedure, first deflating the colon with a colostomy and then revitalizing the patient before definitive surgery, has been beneficial to the improvement in results in patient’s ages 80 years and over.

This study is limited by the retrospective character of the study and the fact that it concerns a single-center study. However, for analyzing the effect of the introduction of a quality improvement program, a homogenous patient group from a single center is more suitable, with a relatively large series and little missing data. Almost all remaining factors were consistent during the timeframe of this study. Obviously, the effect of a learning curve can not be excluded, but over the years, there was no change in the surgical team consisting of all dedicated colorectal surgeons with over 3 years of experience before the introduction of the quality program. In addition, the patient inflow remained consistent, and there were no other changes in postoperative patient management.

It is also acknowledged that in any non-randomized study, even when experimental and control groups appear comparable at baseline, the effect size estimate is still at risk of bias due to residual confounding. One of the largest confounding factors for this study is the concept that results might improve over time due to the surgical learning curve of each surgeon. However, as mentioned, surgeons remained consistent and even when laparoscopic versus open procedure was included in the multivariate analysis, the decrease in major complications after introducing the program remained significant.

## Conclusion

A significant decrease in major complication rates and mortality was observed after careful re-evaluation of national auditing results and introducing this quality improvement program.

This study showed that these relatively easy interventions could be safely implemented and be promising for other hospitals.
